# Reward prospect affects strategic adjustments in stop signal task

**DOI:** 10.3389/fpsyg.2023.1125066

**Published:** 2023-03-17

**Authors:** Valentina Giuffrida, Isabel Beatrice Marc, Surabhi Ramawat, Roberto Fontana, Lorenzo Fiori, Giampiero Bardella, Sabrina Fagioli, Stefano Ferraina, Emiliano Brunamonti, Pierpaolo Pani

**Affiliations:** ^1^Department of Physiology and Pharmacology, Sapienza University, Rome, Italy; ^2^Behavioral Neuroscience PhD Program, Sapienza University, Rome, Italy; ^3^Department of Occupational and Environmental Medicine, Epidemiology and Hygiene, INAIL, Rome, Italy; ^4^Department of Education, University of Roma Tre, Rome, Italy

**Keywords:** stop signal task, cognitive control, inhibitory control, reward, strategic adjustments, motor control

## Abstract

Interaction with the environment requires us to predict the potential reward that will follow our choices. Rewards could change depending on the context and our behavior adapts accordingly. Previous studies have shown that, depending on reward regimes, actions can be facilitated (i.e., increasing the reward for response) or interfered (i.e., increasing the reward for suppression). Here we studied how the change in reward perspective can influence subjects’ adaptation strategy. Students were asked to perform a modified version of the Stop-Signal task. Specifically, at the beginning of each trial, a Cue Signal informed subjects of the value of the reward they would receive; in one condition, Go Trials were rewarded more than Stop Trials, in another, Stop Trials were rewarded more than Go Trials, and in the last, both trials were rewarded equally. Subjects participated in a virtual competition, and the reward consisted of points to be earned to climb the leaderboard and win (as in a video game contest). The sum of points earned was updated with each trial. After a learning phase in which the three conditions were presented separately, each subject performed 600 trials testing phase in which the three conditions were randomly mixed. Based on the previous studies, we hypothesized that subjects could employ different strategies to perform the task, including modulating inhibition efficiency, adjusting response speed, or employing a constant behavior across contexts. We found that to perform the task, subjects preferentially employed a strategy-related speed of response adjustment, while the duration of the inhibition process did not change significantly across the conditions. The investigation of strategic motor adjustments to reward’s prospect is relevant not only to understanding how action control is typically regulated, but also to work on various groups of patients who exhibit cognitive control deficits, suggesting that the ability to inhibit can be modulated by employing reward prospects as motivational factors.

## 1. Introduction

In everyday life, it is important to behave appropriately in relation to the shifting situations we encounter and the effects of our choices. This requires, for example, assessing the potential reward that could be obtained in a specific context before deciding whether or not to act, and being able to stop quickly if sudden changes in the environment make the action no longer useful. A widely used experimental paradigm to study choice behavior is the Stop-Signal Task (SST) in both human and non-human primates (Vince, 1948; Logan and Cowan, 1984; Hanes and Carpenter, 1999; Scangos and Stuphorn, 2010; Brunamonti et al., 2012; Pani et al., 2014; Montanari et al., 2017; Schall et al., 2017; Pani et al., 2018; Bardella et al., 2020; Fiori et al., 2020; Giarrocco et al., 2021; Andujar et al., 2022; Hervault et al., 2022; Pani et al., 2022; Bardella et al., 2023; Marc et al., 2023). The SST requires the subjects to respond as quickly as possible when a Go Signal is presented (for example by pressing a button) and to interrupt the response when unpredictably a Stop Signal is presented after a variable time from the Go Signal. In its original conception, this task creates a competition between two processes: the Go process, triggered by the Go Signal, and the Stop process, triggered by the Stop Signal (race model, Logan and Cowan, 1984). The competition is evident especially in Stop Trials, when first the Go Signal and then the Stop Signal are presented. The choice of moving or stopping that the subject makes will depend on the speed of each of the processes (Logan and Cowan, 1984; Boucher et al., 2007), and on the delay between the Go Signal and Stop Signal (Stop Signal Delay, SSD). The race model suggests that the higher these SSDs are, the greater the probability of moving despite the presentation of the Stop Signal. Effects of choice competition are also present in the trials where only the Go Signal is presented. Indeed, the Go process is affected when subjects know that a Stop Signal is possible as demonstrated by the observation that the same Go responses in tasks without Stop Trials are faster (Mirabella et al., 2008; Pani et al., 2013).

The dual-task nature of the paradigm makes it possible to study two control strategies that support behavior in accordance with the dual-mechanisms framework (DMC): proactive control and reactive control (Braver et al., 2007; Braver, 2012). Correct performance in Stop Trials often requires reactive control, as it allows us to quickly inhibit or reorganize our actions when an unexpected event occurs, rendering planned actions no longer appropriate. The SST provides a measure of this control corresponding to the latency of the reactive inhibition process, i.e., the Stop Signal Reaction Time (SSRT). However, proactive control is also necessary to achieve optimal performance in the task. It allows us to reach a balance between the demand of the task (i.e., responding as quickly as possible), and making as few mistakes as possible on Stop Trials (i.e., optimize the inhibition process). In the SST, for example, the subjects tend to procrastinate their responses as a Stop Signal could possibly be presented. Depending on the context, both control processes can be affected by different factors, such as attention, reward, and motivation (Engelmann and Pessoa, 2007; Mohanty et al., 2008; Engelmann et al., 2009; Krebs et al., 2009; Pessoa and Engelmann, 2010; Padmala et al., 2011; Stoppel et al., 2011; Marc et al., 2023).

Despite thorough exploration of various features of inhibition processes, few studies have investigated how reward can influence motor control, and whether the influence of reward varies depending on how the reward is manipulated. Some studies have manipulated the reward only in the Stop Trials (e.g., Boehler et al., 2012, 2014; Verbruggen and McLaren, 2018). For example, Boehler et al. (2012, 2014), found that SSRTs were shorter when the Stop Signal was associated with a reward for the correct trials, compared to when no reward was associated. However, other studies have not found the evidence to confirm this effect (Schevernels et al., 2015; Verbruggen and McLaren, 2018). In further investigations, the tasks were administered in blocks, and the subjects were informed *a priori* about the reward they would receive for the Correct Stop Trial (Leotti and Wager, 2010; Greenhouse and Wessel, 2013). In these studies, subjects adopted strategies pertaining to the lengthening of RTs, to improve their ability to inhibit. However, when only Go Trials were rewarded, response inhibition was impaired (Padmala and Pessoa, 2010).

To extend this line of research, we investigated how reward perspective can influence one or both controls, reactive and proactive. To this aim, we administered a modified SST in which three different proportions of reward values were randomly presented by informing the subjects about them at the beginning of each trial. Subjects could gain a higher reward (points in a virtual game) for correct Go Trials than for correct Stop Trials, or the same amount of reward for both correct Go and Stop trials, or even a higher reward for correct Stop Trials than for correct Go trials. We found that the subjects employed a strategy based on the modulation of the response’s speed to the Go signal, while the duration of the inhibition process remained unaffected, therefore showing that the behavioral adjustment relied on proactive control.

## 2. Materials and methods

### 2.1. Subjects

We estimated *a priori* the sample size of 14 subjects, on the basis of power 0.90 to detect an effect size in a within-subject design of 0.42 based on partial eta squared (η^2^_p_) 0.15 using GPower 3.1.9.7 (Faul et al., 2007, 2009), as reported in the previous studies employing similar tasks(Mirabella et al., 2008; Andujar et al., 2022; Marc et al., 2023). Eighteen subjects (4 males & 14 females, mean age = 26.5) were recruited for the study. All the subjects were right-handed, had normal or corrected vision, and had no history of psychiatric or neurological disorders.

All subjects were checked for race model violations and respect the unimodality assumption for Stop Signal Delay (SSD) in the Stop Trials prior to testing. Four participants out of 18 were excluded because they did not respect the unimodality assumption for Stop Signal Delay (SSD) in the Stop Trials (see methods for details). All procedures were performed in accordance with the Declaration of Helsinki and after obtaining a written informed consent from each subject. The procedure was approved by the Ethics Committee of “Roma Tre” University.

### 2.2. Apparatus and task

**Figure 1:**
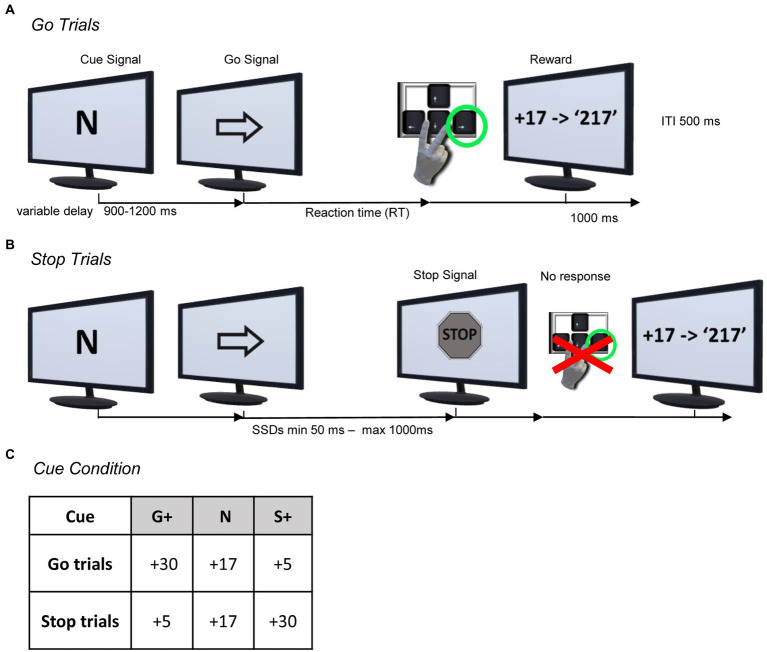
Behavioral paradigm. **(A,B)** Modified version of the Stop-Signal Task (sequence of events for the Neutral Condition; i.e. Cue: N). Each trial started with the Cue Signal, which indicated the type of reward condition to the subject; i.e., the amount of the reward they should expect if they correctly performed the Go Trial (see **A**) or a Stop trial (see **B**). After a variable delay, a Go Signal (right or left arrow) appeared and the subject had to press the arrow key on the keyboard according to the Go Signal to receive the reward as points (see **A**)*.* Occasionally, at a variable delay (SSD) from the Go Signal, a Stop Signal appeared and the subject had to inhibit the action to receive the reward (see **B**). **(C)** Overview of the Cue Signals associated with the respective rewards for each Cue Condition. The amount of reward for every Cue Condition was differently associated with Go and Stop Trials as follows: Go+ Condition (G+, as Cue Signal) was associated with higher reward for successful Go Trials compared to successful Stop Trials; Stop+ Condition (S+, as Cue Signal) was associated with reversed reward amount; Neutral Condition (N, as Cue Signal) was associated with equal amounts of reward for successful Go or Stop Trials.

Subjects were seated in a darkened and sound attenuated chamber, in front of a 20-inch monitor (LCD, 1920 × 1,080 resolution, 60 Hz) placed 40 cm away. Stimuli presentation and behavioral events collection were controlled using the MATLAB-Psychophysics Toolbox Version 3 (PTB-3). They performed a modified version of the Stop Signal Task (Logan and Cowan, 1984; Logan, 1994). As in the classical SST, they were required to perform two types of trials, Go Trials (the majority of trials, 70%) and Stop Trials (the minority of trials, 30%), but presented under three different experimental conditions, indicated by a Cue Signal, (i.e., G+, S+ or N) presented at the beginning of each trial (Figure 1).

In *Go Trials*, after a variable time (900–1,200 ms) from the Cue Signal, a Go Signal (left or right pointing arrow) was presented. The Go Signal required the subjects to press the left or right arrow key on the keyboard as fast as possible with their right index or middle finger, indicated by the presented Go Signal, within an upper reaction time of 1.5 s. If the subject responded correctly (i.e., Correct Go Trial), visual and positive auditory feedback (1 s) were presented with the score obtained in relation to the trial condition.

If the subject did not respond correctly (Failed Go Trial) by failing to press the corresponding key to the Go Signal or not responding within the upper reaction time (Go omission) after the Go Signal, visual feedback, “*0 Score*” or “*0 Score, too late!*,” respectively, was provided along with a negative audio feedback.

In *Stop Trials*, the initial sequence of events was identical to that of the Go Trials, but after a variable time from the Go Signal presentation (Stop Signal Delay [SSD]), a Stop Signal appeared, which required subjects to inhibit the movement triggered by the Go Signal. If the subject withheld the movement correctly (Correct Stop Trial), after the disappearance of the Stop Signal (1 s.), a visual and positive auditory feedback (1 s.) appeared with the score obtained in relation to the trial condition. If the subject did not respond correctly (Failed Stop Trial), by failing to inhibit the movement, visual feedback, “*0 Score*,” was provided to the subjects along with a negative audio feedback.

In the Stop Trials, a staircase procedure adjusted the SSD based on subjects’ performance. The SSD started at an initial value of 50 milliseconds (ms), and the SSDs following Correct Stop Trials were increased by one step (50 ms) while SSDs following Error Stop Trials were decreased by one step in the next Stop Trial. The minimum and maximum limits of the SSD presentation were set to 50 milliseconds and 1 s, respectively. The intertrial interval was 500 ms.

This method keeps the probability of error at the presentation of the stop signal around 50%, deterring the subject from using strategies in which he is expected to wait for the stop signal (Verbruggen et al., 2019).

The two types of trials, Stop and Go Trials, were presented with the same proportions (i.e., 30% Stop Trials and 70% Go Trials) in the three different experimental conditions in mixed order.

*Go + Condition*. In the Go+ condition, subjects were informed that performing Correct Go Trials would allow them to earn a higher reward (+30 points), whereas Correct Stop Trials would allow them to earn a lower reward (+5 points).

*Stop + Conditions*. On the contrary, in the Stop+ condition, subjects were informed that Correct Go Trials would allow them to earn a lower reward (+5 points), whereas Correct Stop Trials would allow them to earn a higher reward (+30 points).

*Neutral Conditions*. In the Neutral condition, the amount of reward subjects could earn was the same in Correct Go Trials and Correct Stop Trials (+17 points).

For all the conditions, the positive auditory feedback was the same but differed in pitch according to the amount of reward the subject received upon the completion of a correct trial (i.e., high frequency = +30 scores; low frequency = +5 score).

### 2.3. Experimental procedure

Subjects executed the three different experimental Cue Conditions in two phases, a learning phase and a testing phase.

#### 2.3.1. Learning phase

Each subject performed separately, in blocks of 200 trials, the three conditions with an independent staircase procedure for the presentation of the SSDs. The order in which the blocks were presented was randomized and counterbalanced between all subjects.

#### 2.3.2. Test phase

One week after the learning phase, each subject performed a single block of 600 trials comprising the three conditions in a mixed order, with an equal probability of presentation and randomized trial-by-trial. In this way, the subjects became aware of which reward they would earn at each trial only at the presentation of the Cue signal. In the test phase, 1/3 of the trials (Go and Stop Trials) were designated to the Go+ condition, 1/3 to the Neutral condition and 1/3 to the Stop+ condition. Within this phase, three independent staircases for the three conditions were used.

#### 2.3.3. Instructions

Before each block, subjects were instructed to perform the task in that specific condition, incentivizing them to respond as quickly as possible and at the same time to be ready to stop at the appearance of the Stop Signal to earn as many rewards (points) as possible to outperform the other subjects. Additionally, the staircase procedure was also explained to subjects to further discourage a waiting strategy (see Verbruggen et al., 2019). Immediately afterwards, an actual and updated ranking of the previous subjects’ scores for that condition was shown, inviting the subject to beat the current record. Except for the first subject, who was shown the highest score attainable with the best performance in the task.

### 2.4. Data analysis

Since the Learning Phase served only for the subjects to become familiar with the three Cue Conditions and to understand the experimental procedures, the reported analyses were performed only on the Test Phase.

To verify whether there was an effect of the Cue Condition on the subject’s strategy across the conditions, we considered different variables as follows: (1) the reaction time (RT) in Go Trials; (2) the probability of Go omissions; (3) the probability of response [p(response)] in Stop Trials (Failed Stop); (4) the average SSD and (5) Stop Signal Reaction Time (SSRT). The RTs in Go Trials were calculated as the time between the appearance of the Go signal and the subjects’ response. All subjects’ performances were checked for compliance with assumption of independence of race model and unimodality tests for SSDs in the Stop Trials prior to further analyses. Hartigan’s dip test statistic for unimodality was applied on RTs and SSDs (Hartigan, 1985; Hartigan and Hartigan, 1985). In order to test for significance, bootstrap was set at 1,000.

Four subjects were excluded because they did not respect the unimodality assumption for SSD in the Stop Trials (Mean = 0.08; *p* < 0.05).

Then, the probability of Go omission was calculated as the number of Go omissions divided by the total number of Go Trials. The probability of response in Stop Trials (Failed Stop) was calculated as the number of Failed Stop Trials divided by the total number of Stop Trials. SSRT was calculated using the integration method (with replacement of Go omission) (see Verbruggen et al., 2019), after verifying that none of the subjects had violated the horse race model’s assumption of independence, and the p(response) in Stop Trials was between 35 and 65%. All variables of interest were computed for each subject individually and for each Cue Condition in the Test Phase. To test whether the three Cue Conditions influenced the subjects’ strategy, comparisons between Cue Conditions and within subjects were performed by repeated measures factorial ANOVAs, followed by post-hoc comparisons by Tukey–Kramer test. To assess how typical or uncommon the effect measured within the population mean is we applied Bayesian analysis (Ince et al., 2021, 2022). Bayesian prevalence returns a posterior distribution over the population prevalence, given the observed distribution of RTs for each Cue Condition. From this, we computed the maximum a posterior (MAP) estimate, that is the value of the population parameter. To quantify the uncertainty of this estimate, calculation of Bayesian highest posterior density intervals (HPDIs) allowed us to have a range within which the true population value lies with the specified probability.

Finally, we investigated with an exploratory approach which internal component (Luce, 1986; Ratcliff, 1988) could explain the difference between the conditions highlighted in the RTs distribution by fitting the drift diffusion model (DDM) to the data through DMAT toolbox (Vandekerckhove and Tuerlinckx, 2007, 2008).

Following this model, the decision process is represented as an accumulation of evidence toward a decision criterion represented by a boundary. More specifically, subjects start to accumulate sensory evidence at an initial point (*z*) until the process reaches a boundary [or threshold (*a*)], and the response (choice) is generated. There are diverse parameters that can explain the features of the RTs distributions observed. Specifically: (a) the boundary separation (*a*), that is the distance between two boundaries, one corresponding to the correct responses and the other to wrong responses; b) the starting point of the evidence accumulation process (*z*); (c) the rate of growth toward the boundary [drift rate (*v*)]; (d) the duration of the non-decision process (T*er*); (e) the variability in starting point (*sz*); (f) the variability in non-decision time (*st*); (g) and the variability in stimulus quality (*η*) (Ratcliff, 1979, 2002; Voss et al., 2004; Ratcliff and McKoon, 2008).

In our investigation we postulate that the accumulation process (i.e., decision process) in our task is influenced by the Cue signal which, by providing information about the reward schedule, introduces a choice bias before the presentation of the Go signal (Ratcliff, 1985; Carpenter and Williams, 1995; Gold and Shadlen, 2002; Ratcliff and McKoon, 2008; Lauwereyns, 2011). Previous studies have identified two ways in which *a priori* information can bias the decision process: by shifting the starting point for the accumulation process of sensory information over time to a decision threshold (Edwards, 1965; Link and Heath, 1975; Ratcliff, 1985; Voss et al., 2004; Bogacz et al., 2006; Diederich and Busemeyer, 2006; Wagenmakers et al., 2008); or by changing the drift rate, i.e., the speed at which sensory information accumulates over time (Ashby, 1983; Ratcliff, 1985; Diederich and Busemeyer, 2006).

Both models would generate faster RTs in the Go+ condition and slower RTs in the Stop+ condition.

We therefore fitted the DDM to the RTs from each subject and each condition (Go +, Stop +, Neutral) with three different models. In the first both drift rate(v) and starting point(z) varied between conditions. In the second and the third either the drift rate(v) only, or the starting point(z) only, varied.

The RTs data were divided into percentiles, and we used a chi-square (*χ*^2^) percentile-based method as an estimation method. The relative fit of the models was tested as follows: the difference in BIC scores was calculated for each individual subject and model and the model with the lowest BIC was found to be a better fit to the data.

Finally, we applied the non-parametric Friedman’s test to assess whether the estimated parameters were different between conditions.

The RTs data were divided into percentiles, and we used a multinomial Likelihood percentile-based method as estimation method. Finally, we applied the non-parametric Friedman’s test to assess whether the estimated parameters were different between conditions.

Data processing and analysis were performed by custom functions developed in Matlab.^1^

## 3. Results

### 3.1. Influence of reward on reaction time

**Figure 2:**
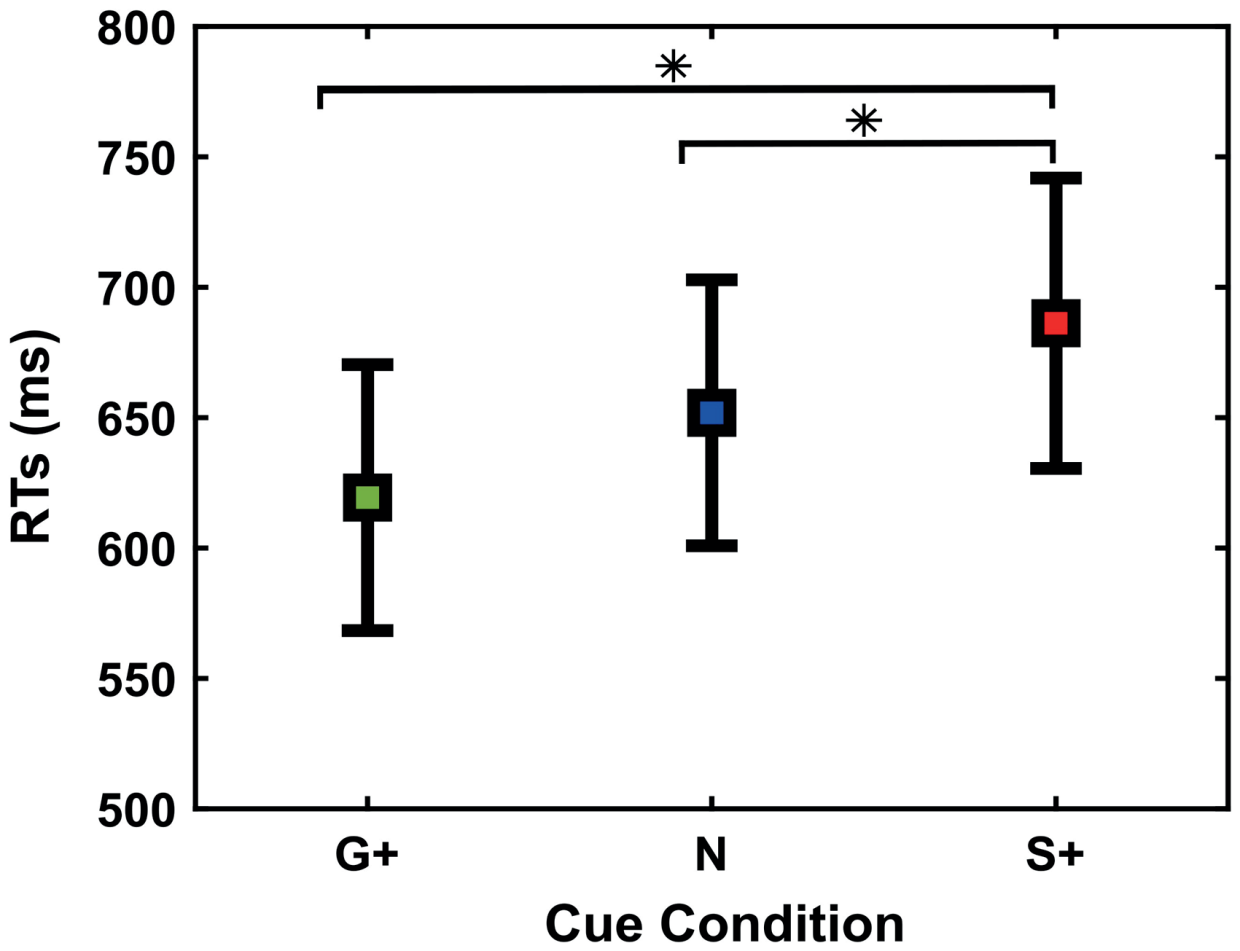
Reaction times [RTs (ms)] (mean and ± 1SEM) of Go Trials in the different cue conditions (G+, N, S+).

One of the aims of the experiment was to assess how the reward context could modulate the strategy used by the subjects in order to execute or inhibit their movement. Therefore, we first tested whether the RTs changed between Cue Conditions using repeated measures ANOVA (Figure 2).

We found that subjects adjusted RTs according to the Cue Conditions [Go+ Condition (Mean = 619 ms, SD = 180 ms, SEM = 48 ms); Neutral Condition (Mean = 652 ms, SD = 191 ms, SEM = 51 ms); Stop+ Condition (Mean = 686 ms, SD =208 ms, SEM = 56 ms)], *F*(2,26) = 9.71; *p* < 0.001.

Post-hoc analysis shows that the responses in both Go+ and Neutral Conditions were faster than responses in Stop+ Condition, *p* = 0.01 and *p* = 0.02. This suggests that the subjects were slower in responding in the Stop+ Condition, in which a higher reward was provided for Correct Stop Trials, while no significant difference was found between RTs in the Neutral and Go+ Condition, *p* = 0.07.

### 3.2. Influence of reward condition on inhibition strategy

**Figure 3:**
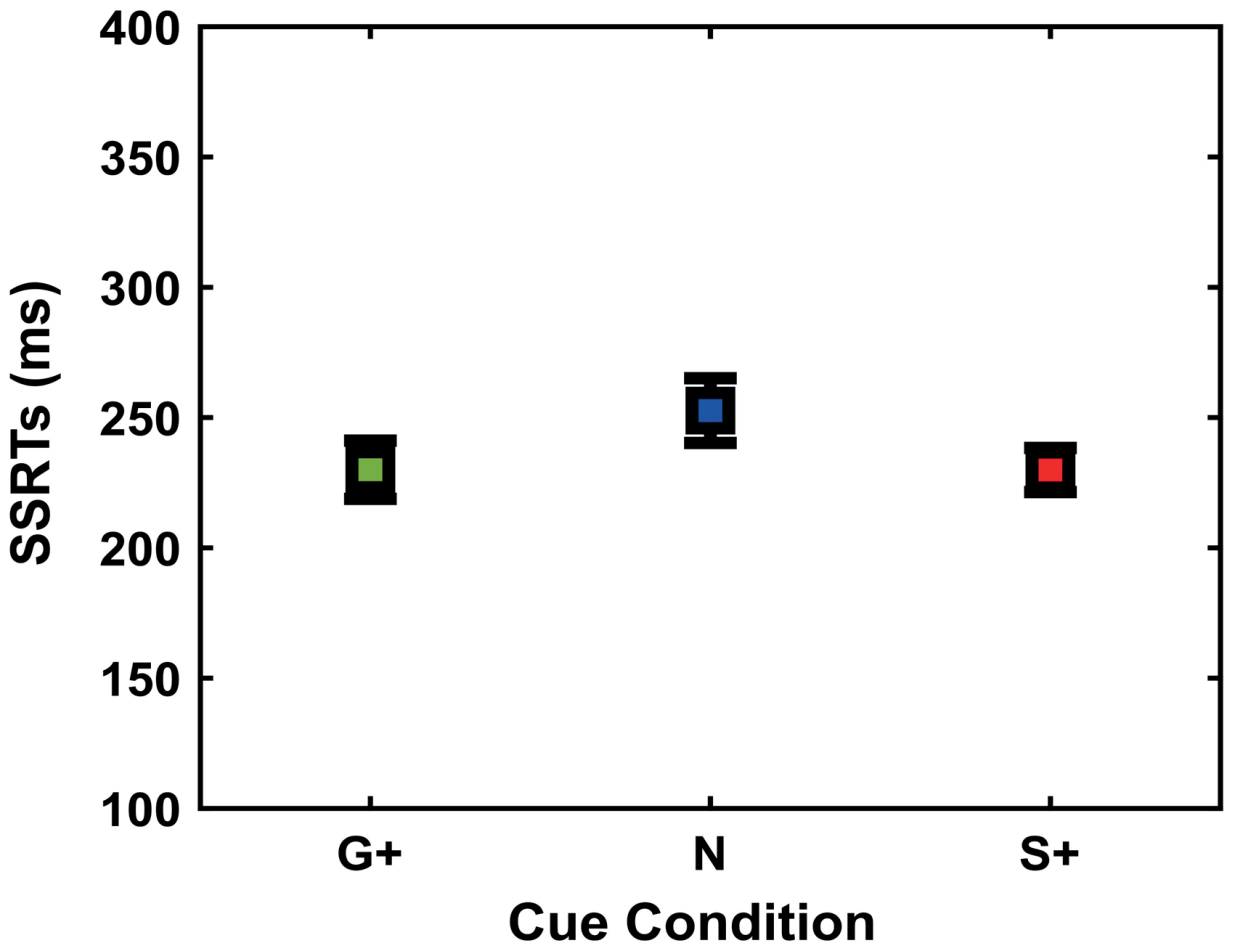
Stop signal reaction time [SSRT (ms)] (mean and ± 1SEM) estimated in the different cue conditions (G+, N, S+).

To assess whether the reward prospects exerted an influence on the subjects’ inhibition ability, for each Cue Condition we analyzed three variables: SSRTs, mean of SSDs, and p(response) to the Stop Signal. First, repeated measures ANOVA was used to analyze SSRTs between conditions [Go+ Condition (Mean = 230 ms, SD = 41 ms, SEM = 11 ms); Neutral Condition (Mean = 253 ms, SD = 46 ms, SEM = 12 ms); Stop+ Condition (Mean = 230 ms, SD = 32 ms, SEM = 8 ms)], as it measures the time it takes for each subject to successfully interrupt the action in progress (Figure 3). Results showed no significant differences between conditions, *F* (2,26) = 3.17; *p* = 0.058.

**Figure 4:**
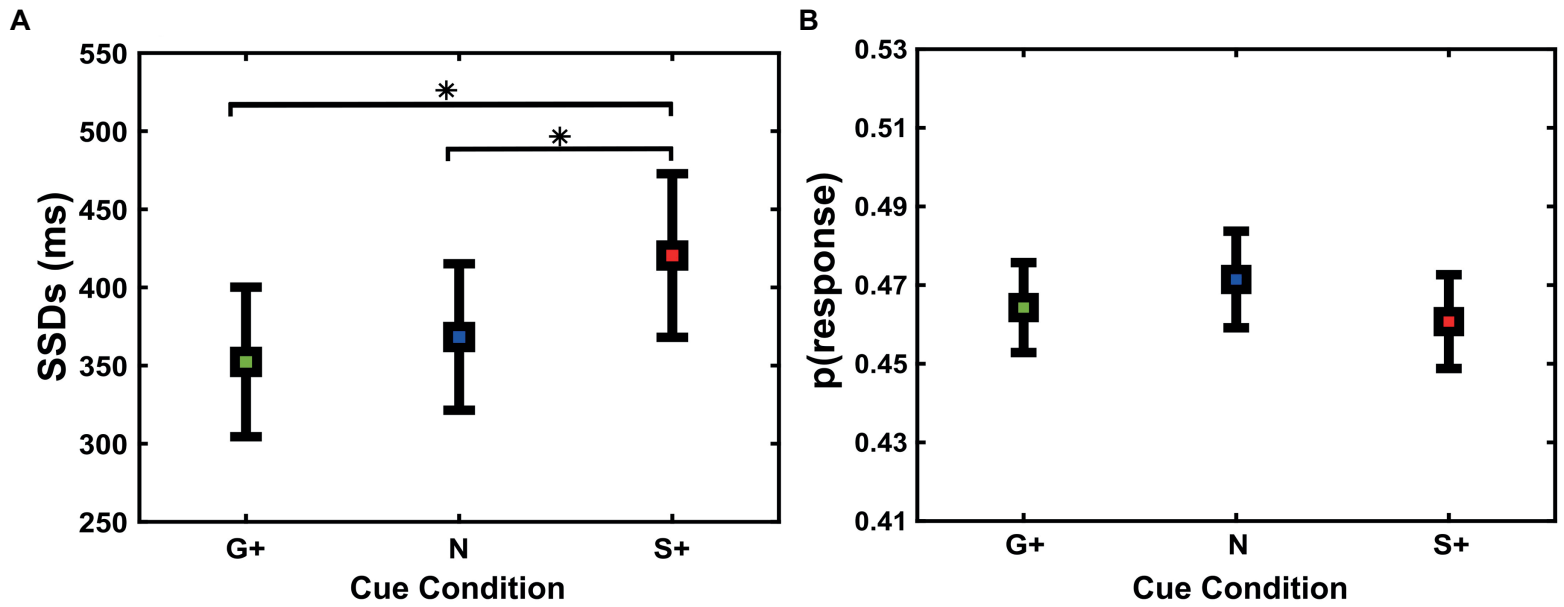
**(A)** Average stop-signal delays (SSD) (mean and ± 1SEM) and **(B)** probabilities to respond to the stop-signal [p(response)] (mean and ± 1SEM) as a function of cue conditions (G+, N, S+).

We also tested another closely related behavioral marker dependent on the inhibition process, namely the SSD, between Cue Conditions [Go+ Condition (Mean = 352 ms, SD = 179 ms, SEM = 48 ms); Neutral Condition (Mean = 368 ms, SD = 175 ms, SEM = 46 ms); Stop+ Condition (Mean = 420 ms, SD = 195 ms, SEM = 52 ms)]. Specifically, SSDs can provide crucial insights into behavioral adjustments through the inhibition task (van Boxtel et al., 2001; Band et al., 2003). Repeated measures ANOVA analysis showed a main effect of Cue Conditions (Figure 4A) on SSDs *F*(2,26) = 8.45; *p* = 0.001.

Post-hoc analyses revealed that SSDs were significantly longer when correct performance was rewarded more in Stop Trials, i.e., Stop+ Condition, than at the Neutral and Go+ Condition (*p* = 0.01; *p* = 0.03, respectively), and no significant difference was found between SSDs in the Go+ and Neutral Condition (*p* = 0.6). Despite the increase in SSDs in the Neutral and Stop+ Conditions, subjects did not show significant differences in p(response) on Stop Trials (Figure 4B), *F*(2,26) = 0.46; *p* = 0.6, nor in the probability of Go omission, *F*(2,26) = 2.19; *p* = 0.1. These results suggest that the increase in SSDs between Cue Conditions shows a greater capacity for inhibition in subjects when correct Stop Trials were rewarded more than correct Go Trials.

### 3.3. Proactive strategic adjustment to the reward conditions

**Figure 5:**
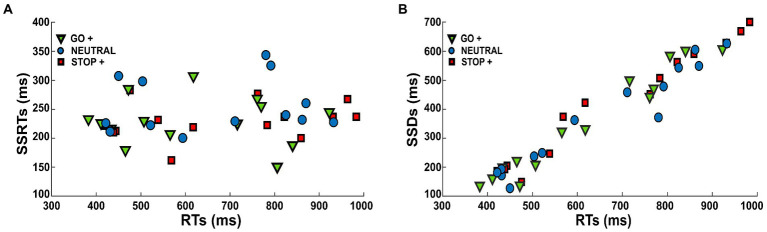
Scatter diagrams show **(A)** RTs as a function of SSRTs and **(B)** RTs as a function of SSDs in each Cue Condition.

Our analysis suggests that subjects coped with the task by lengthening their RTs according to the different Cue Conditions, while their probability of response (p(response)) and inhibition speed (SSRTs) were kept constant. These data suggest that the subject adopted a proactive strategy. If this was the case, we should expect that the SSDs changed accordingly to the RTs. Indeed, by slowing down their RTs, subjects should be able to inhibit at the same speed (SSRT), and at the same probability (p(response)) at longer SSDs. To test this hypothesis, we estimated the correlation between RTs and SSRTs in the different Cue Conditions (Figure 5A), and we found that there was no correlation (Pearson correlation, *r* ***<*** 0.25; *p* > 0.5). We then tested the correlation between RTs and SSDs in the different Cue Conditions (Figure 5B), and we found a significant positive correlation (Pearson correlation, *r* > 0.97, *p* < 0.001).

### 3.4. Prevalence of the effects in the population

**Figure 6:**
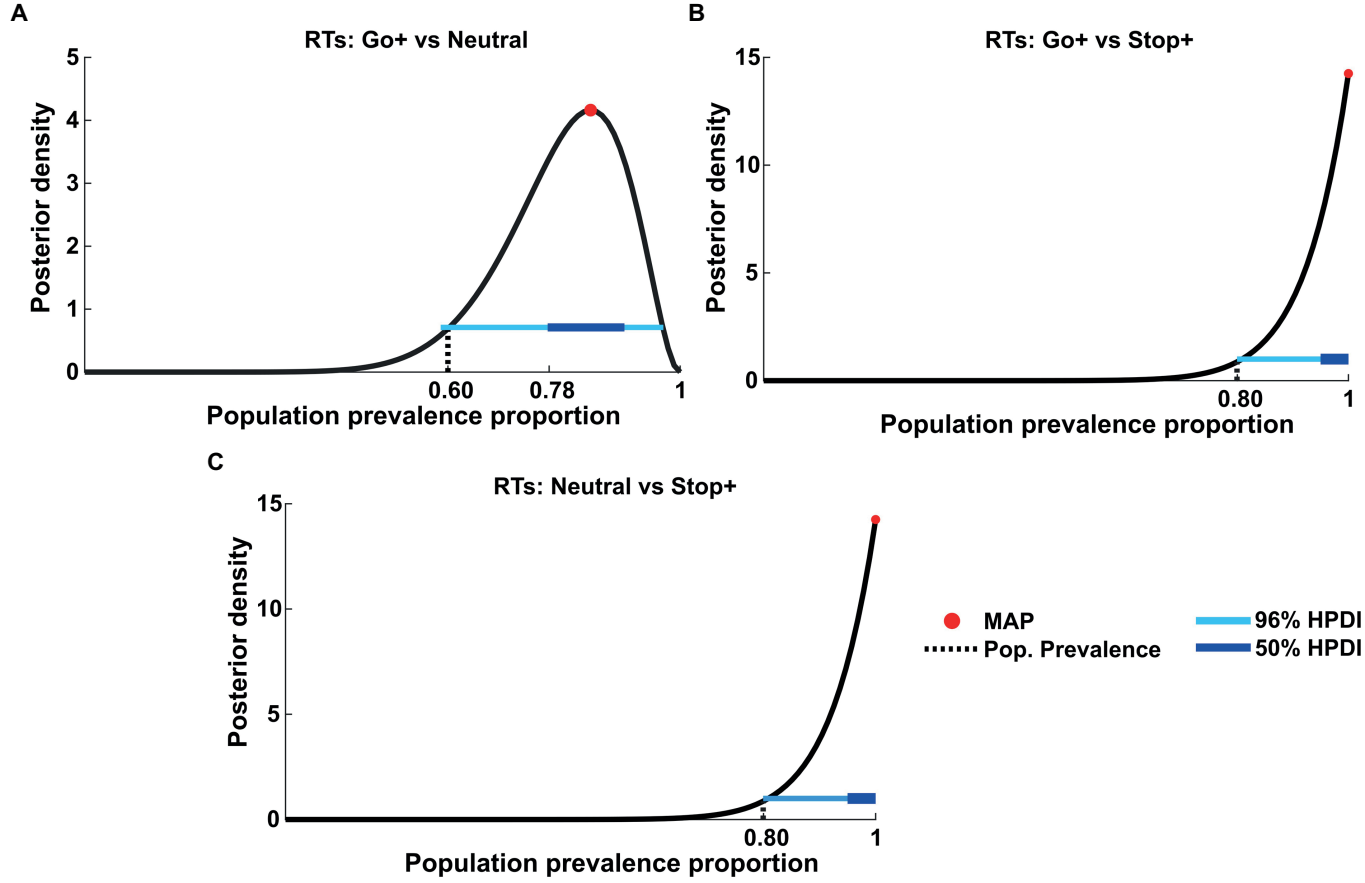
Bayesian inference of population prevalence for RTs. Each panel shows posterior density (black curve) with MAP (red circle), 50% and 96% HPDI (thick/thin blue lines), obtained from within-subject inference at *p* = 0.05 of RTs for each Cue Condition interaction: **(A)** RTs tested on Go+ Condition versus Neutral Condition; **(B)** RTs tested on Go+ condition versus Stop+ Condition; **(C)** RTs tested on Stop+ Condition versus Neutral Condition.

Once we established that the results support the adoption of a proactive strategy to perform the task, we tested the prevalence in the population of the main effects we found. To this aim, we employed Bayesian inference by applying t-tests to paired within-subject samples at the first level to compare the distribution of RTs between Cue Conditions (Figure 6).

The population prevalence proportion of the within-subject t-test between RTs in the Go+ and Neutral Cue Conditions (Figure 6A) showed the MAP estimate of prevalence to be 0.85 (96% HPDI: [0.59 0.97]), whereas RTs in the Go+ vs. Stop+ (Figure 6B) and Stop+ vs. Neutral (Figure 6C) Conditions showed the MAP estimate of prevalence to be equal 1 (96% HPDI: [0.80 1]).

Based on this result, we concluded that it is highly probable that more than 59% of the population would show a true significant effect when comparing the Go+ condition to the Neutral condition, and we would also consider it highly probable that the result obtained between the Go+ vs. Stop+ and Stop+ vs. Neutral RTs would recur at 80% when tested in the same experiment.

**Figure 7:**
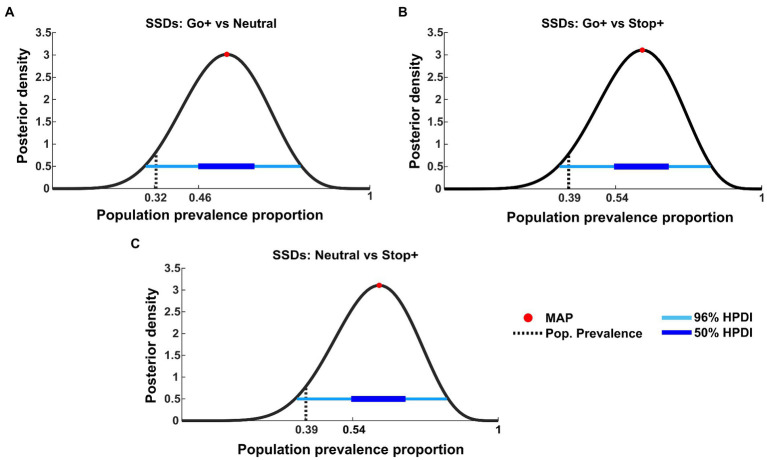
Bayesian inference of population prevalence for SSDs. Each panel shows posterior density (black curve) with MAP (red circle), 50% and 96% HPDI (thick/thin blue lines), obtained from within-subject inference at *p* = 0.05 of RTs for each Cue Condition interaction: **(A)** SSDs tested on Go+ Condition versus Neutral Condition; **(B)** SSDs tested on Go+ Condition versus Stop+ Condition; **(C)** SSDs tested on Stop+ Condition versus Neutral Condition.

We also tested the effect found for SSDs. Similarly, we applied the t-tests to paired within-subject samples at the first level to compare the SSD distribution between conditions (Figure 7).

The effects found in SSDs are not as strong as those shown in RTs. In fact, this analysis shows that if other subjects were to be tested in the same experiment, the probability of obtaining the same results is possible between 32% (MAP = 0.55, 96% HPDI: [0.28 0.79], 50% HPDI: [0.46 0.64], Figure 7A) and 39% of the tested subjects (MAP = 0.62, 96% HPDI [0.35 0.85], 50% HPDI: [0.46 0.64], Figures 7C,D).

These data integrate the previous findings by showing that despite the subjects employing the strategy of elongating the RTs when higher reward is provided for Stop than for Go trials, some change in the speed of inhibition can also be at play. In fact, as also shown by the SSRT results, the tendency to decrease SSRTs in the Stop+ Condition, could indicate that for some subjects the higher reward for Stop Trials can also lower SSRT.

### 3.5. Fitting the drift diffusion model to the RTs distribution

**Figure 8:**
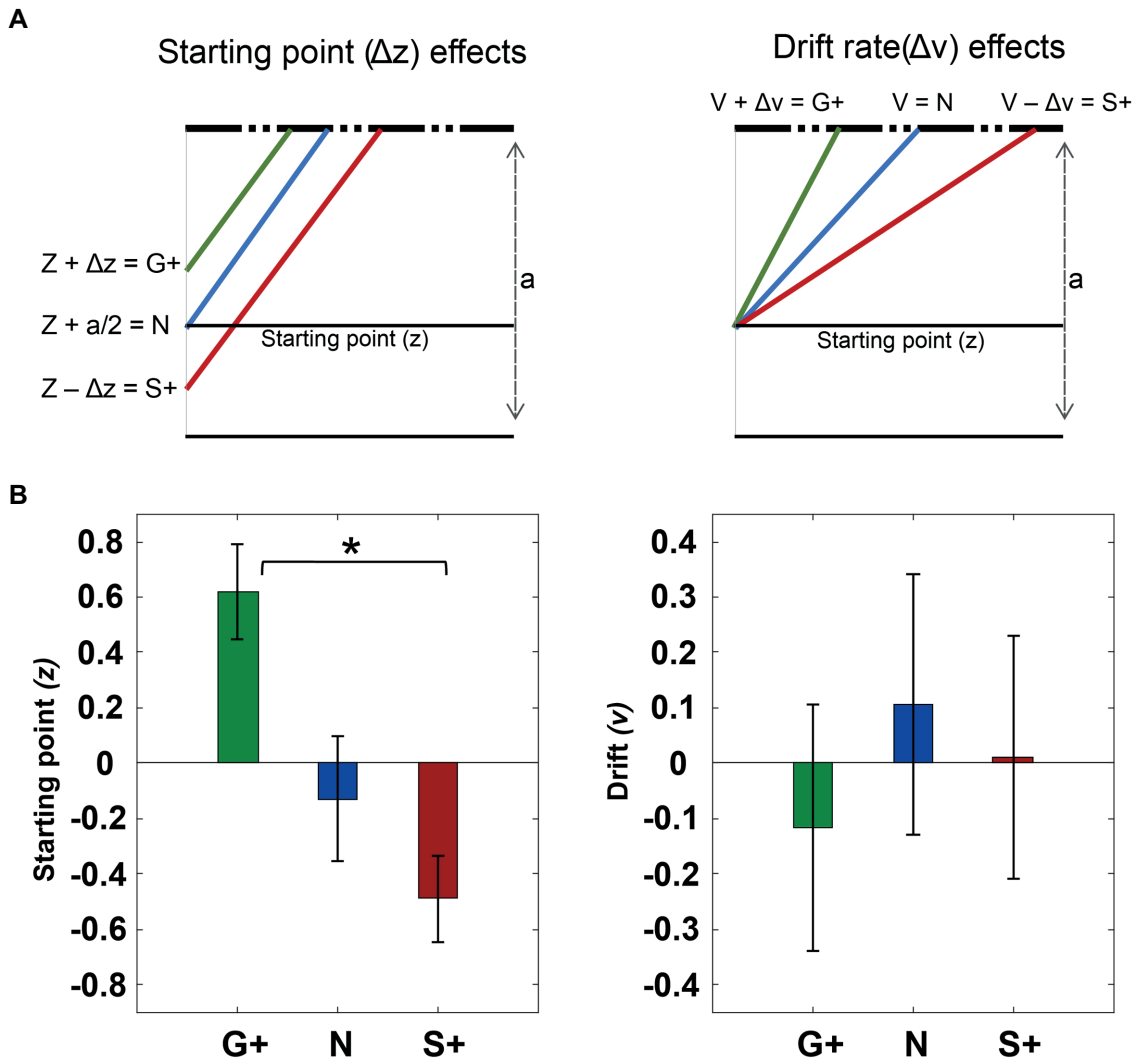
Fit data to drift diffusion model (DDM). **(A)** Fit data to drift diffusion model (DDM). **(A)** Effects explained with DDM. the effect at the starting point z, we assume that it is close to the boundary in the Go+ condition, in the middle of the threshold (a) in the neutral condition, and finally moving away from the correct boundary in the Stop+ condition. Similarly in the drift model (v), we assume that it increases for the Go+ condition and decreases in the Neutral and Stop+ conditions. **(B)** The zscore average of the effect of the starting point parameter (z) and the zscore average of the parameters of the drift rate(v) (right panel). Error bars represent 1 SE from the mean.

We evaluate different models to investigate which parameters of the DDM could explain the changes in RTs distributions associated with the different reward conditions. Specifically, in the Go+ condition the shortening of RTs with respect to the Stop+ condition can be explained by: a higher drift rate (drift rate (v)), i.e. a fast decision process; a higher starting point (starting point (z)) for the decision process, that has been set following the Cue signal; or a modulation of both (Figure 8A). We estimated three different models (model 1 = ‘affect Drift and Starting point’; model 2 = ‘effect only on v’ and model 3 = ‘effect only on z’). We also tested intermediate models to evaluate the goodness of the fit.

The sum of Bayesian information criterion values (BIC; Schwarz, 1978) across subjects show a lower value for the model ‘effect only on z’ than for the drift model (BIC: ‘effect only on v’ = 2.25 + e04; ‘effect only on z’ = 2.25 + e04; ‘effect on drift and z’=: 2.18 + e04). At the individual level, we compared the BICs of the models within each subject for the two model without interaction, and we found that for 10 out of 14 subjects the fitting was better with ‘effect only on z’; and for the others 4 showed better fitting was obtained for the model ‘effect only on v’.

Finally, the mixed model, even at the individual level, had the lowest values of BIC. Therefore, we took this as the model that fitted best with our data.

To assess how the estimated parameters vary between conditions, we applied the Friedman test that shows a significant difference across conditions for the starting point(z) (*p* < 0.01), but no difference was found for the drift rate(v) across conditions. The post-hoc shows a significant difference between the Go+ condition and the Stop + condition (*p* < 0.01), but no difference is present between the Neutral condition and the Go+ (*p* = 0.14) and versus Stop+ (*p* = 0.38) (Figure 8B). To conclude the changes in RTs observed between conditions can be mostly related to changes in the starting point of the accumulation process.

## 4. Discussion

The primary goal of this study was to compare how different reward conditions affect the strategy of control in a complex context. Previous studies have shown that the reward perspective influences cognitive functions, such as working memory (Gilbert and Fiez, 2004; Beck et al., 2010) and attention (Krebs et al., 2009; Padmala et al., 2011; Stoppel et al., 2011; Schevernels et al., 2015). In these studies, the reward availability, compared to conditions when no reward was delivered, enabled an improvement in proactive processes through top-down control (Pessoa and Engelmann, 2010; Chelazzi et al., 2013). The few studies that have investigated the influence of reward on response inhibition have employed paradigms in which the motivation revolved around the presence or absence of reward (Scheres et al., 2001; Greenhouse and Wessel, 2013; Rosell-Negre et al., 2014), or specifically, for response inhibition the type of Stop Signal presented informed about the presence or type of reward (Boehler et al., 2012, 2014; Wilbertz et al., 2014).

In the wake of these studies, we administered a modified SST, in which we introduced different amounts of reward following a dynamic presentation that varied trial by trial, to investigate how reward influenced the proactive and/or reactive strategy on Go and Stop processes. Our results show that the value of the reward has an effect on the RTs of the subjects. Subjects tended to be faster when correctly responding to the Go Signal was rewarded more than correctly performing the Stop Trials, compared to the other context when response inhibition was rewarded more.

Subjects tended to respond faster when the Go trials were rewarded more than the Stop Trials, and vice versa. This is congruent with previous findings, revealing the effects of reward bias on Stop-signal task performance (Leotti and Wager, 2010; Padmala and Pessoa, 2010). Slowing down in the Stop+ versus Neutral and Go+ condition, can be attributed to adjustments in proactive inhibitory control by trading speed at the Go Trial for success at the Stop Trials (Verbruggen et al., 2009; Aron, 2011). These adjustments were performed according to the cues presented at the beginning of the trials.

No significant effect on SSRTs was observed across conditions, which is coherent with other studies (Logan et al., 1986; Ramautar et al., 2004; Lansbergen et al., 2007; Zandbelt et al., 2013; Verbruggen and McLaren, 2018; Andujar et al., 2022). This could be due to the strategy used by the subjects.

In our task a staircase algorithm has been employed to change the SSD according to the performance in Stop Trials. The goal of this procedure was to keep the p(response) as close as possible to 0.50. Therefore, an important index to consider in our study is SSD, and how it changed according to the context to provide information regarding the strategy that the subjects have been using during the task (van Boxtel et al., 2001; Band et al., 2003).

In line with the goal of the staircase procedure, we did not find a difference in p(response) between conditions; however, we found that SSDs correlated positively with RTs, and longer SSDs were attributed to the Stop + condition. This shows that the subjects were able to inhibit the movement at longer SSD by lengthening their RT. Thus, the higher reward for Correct Stop Trials prompted the subjects to slow down their response that, consequently, required the increase of SSD to obtain the p(response) by the staircase algorithm. In this context, we observed only slight changes in the SSRT, supporting the idea that subjects approached the task mostly changing their RT while keeping their speed of inhibition unaffected.

However, our prevalence analysis has shown that SSD changes were not as strong as RT changes. This suggests that the subjects could also have partially adopted another strategy, based on the concurrent modulation of the speed of inhibition. It is indeed possible that in Stop+ trials the subjects have also shortened their SSRT, thus making them able to inhibit at an even longer SSD.

Our results are consistent with the theoretical approach which suggests that adopting a proactive control strategy in reward conditions improves goal attainment (Braver, 2012). In this sense Jimura et al. (2010) argued that the adoption of a proactive control strategy involves the maintenance and preparatory updating of task goals, which facilitates performance in reward contexts.

We investigated the behavioral results by employing the Drift Diffusion Model and we found that in most subjects the choice bias is mainly determined by adjusting the starting point of the accumulation process rather than by changing the accumulation rate itself. This suggests that, at least in this study, proactive control is obtained by adjusting the starting point of the response preparation process following the Go presentation. Thus, following the Stop+ cue, the lowering of the starting point will require more time to reach the boundary producing longer RTs. These findings are in line with results obtained from cortical premotor neuronal recordings in primates performing similar tasks (Giamundo et al., 2021). Indeed, premotor neuronal activity reflects the level of motivation to move before the Go signal: higher activity for the Go+ than for Neutral and Stop+ conditions (see Figure 3 of Giamundo et al., 2021). This modulation is reminiscent of the starting point modulation that we observed. Furthermore, human studies that used model-based approaches to investigate bias in choice behavior show similar results(Ratcliff, 1988; Voss et al., 2004; Palmer et al., 2005; Bogacz et al., 2006; Forstmann et al., 2010; Mulder et al., 2012).

One of the interesting results of the study concerns the Neutral condition. Subjects tended to have RTs whose values were between the RTs of the Go+ and Stop+ Condition but had slightly longer SSDs compared to the Go+ Condition, and higher SSRTs compared to the other two Conditions, thus slightly increasing the p(response) in Stop trials.

It could be argued that this condition implies greater cognitive effort for the subject, because the reward of correct Go and Stop Trials have equal value, implying greater effort to do the two types of trials correctly.

In line with the theory of effort allocation, these results show that subjects are able to improve performance if the task has a high value relative to cognitive effort (i.e., Go+ and Stop+ Condition) and, conversely, performance decreases if the task has a low value relative to task demand(i.e., Neutral condition) (Kurzban et al., 2013; Thomson et al., 2015; Massar et al., 2016).

Instead, in the Go+ and Stop+ conditions, the higher reward and a lower one could be seen by the subject as a reinforcement (+30 points) and a punishment (+5 points, in accordance with the Cue Condition) by shifting the focus to performing one type of trials correctly rather than the other.

Moreover, previous studies suggest such reward perception encourages flexible behavior (Maddox and Markman, 2010), in fact in the Go+ and Stop+ Conditions, the strategy adopted changes and appears to be consistent with receiving the high reward (Verbruggen et al., 2017; Verbruggen and McLaren, 2018).

With these results, subsequent studies could investigate how reward value influences motor control, using electrophysiological measures of cognitive effort to test their relationship. Furthermore, the results obtained through Bayesian analysis show a reproducibility of the SSD-related effect of less than 40%. In order to verify whether a reactive strategy was used more in some subjects, a future study should also include testing the reactive strategy within the task.

## 5. Conclusion

In accordance with previous studies, subjects could employ different strategies to perform the task, including adjusting the speed of response or modulating the efficiency of inhibition. Subjects preferentially adjusted their speed of response, although in some cases a concurrent adjustment of the speed of inhibition could have been at play.

The investigation of strategic motor adjustments based on reward perspectives is relevant not only for understanding how action control is typically regulated, but also for studying genetic underpinnings of control strategies (Mione et al., 2015) and various groups of patients with cognitive control deficits (Brunamonti et al., 2011; Pani et al., 2013; Duprez et al., 2016; Olivito et al., 2017; Menghini et al., 2018). These studies will help to understand how the control processes (proactive and reactive) can be regulated by using reward perspectives as motivational factors.

## Data availability statement

The raw data supporting the conclusions of this article will be made available by the authors, without undue reservation.

## Ethics statement

The procedure was approved by the Ethics Committee of “Roma Tre” University. The patients/participants provided their written informed consent to participate in this study.

## Author contributions

PP and VG conceived the original idea. PP, EB, and SFe supervised the project. VG created the task and collected the data. PP, IM, and VG conceived and performed the data analysis. VG, IM, SR, and PP wrote the paper. All authors contributed to the article and approved the submitted version.

## Funding

This study was partially supported by a Sapienza grant (RM11916B89232364 to PP).

## Conflict of interest

The authors declare that the research was conducted in the absence of any commercial or financial relationships that could be construed as a potential conflict of interest.

## Publisher’s note

All claims expressed in this article are solely those of the authors and do not necessarily represent those of their affiliated organizations, or those of the publisher, the editors and the reviewers. Any product that may be evaluated in this article, or claim that may be made by its manufacturer, is not guaranteed or endorsed by the publisher.

## Footnotes

^1^ https://it.mathworks.com/
